# A Review on Synthesis, Properties, and Applications of Polylactic Acid/Silica Composites

**DOI:** 10.3390/polym13183036

**Published:** 2021-09-08

**Authors:** Mosab Kaseem, Zeeshan Ur Rehman, Shakhawat Hossain, Ashish Kumar Singh, Burak Dikici

**Affiliations:** 1 Department of Nanotechnology and Advanced Materials Engineering, Sejong University, Seoul 05006, Korea; 2 School of Materials Science & Engineering, Changwon National University, Changwon 641-773, Korea; Zeeshan.physics@gmail.com; 3 Department of Industrial and Production Engineering, Jashore University of Science and Technology, Jashore 7408, Bangladesh; shakhawat.ipe@just.edu.bd; 4 Department of Applied Sciences, Bharati Vidyapeeth’s College of Engineering, New Delhi 110063, India; ashish.singh.rs.apc@itbhu.ac.in; 5 Department of Metallurgical and Materials Engineering, Ataturk University, Erzurum 25240, Turkey

**Keywords:** polylactic acid, silica, composites, thermal stability, toughness, biodegradability, 3D printing applications

## Abstract

Polylactic acid (PLA)/silica composites as multifunctional high-performance materials have been extensively examined in the past few years by virtue of their outstanding properties relative to neat PLA. The fabrication methods, such as melt-mixing, sol–gel, and in situ polymerization, as well as the surface functionalization of silica, used to improve the dispersion of silica in the polymer matrix are outlined. The rheological, thermal, mechanical, and biodegradation properties of PLA/silica nanocomposites are highlighted. The potential applications arising from the addition of silica nanoparticles into the PLA matrix are also described. Finally, we believe that a better understanding of the role of silica additive with current improvement strategies in the dispersion of this additive in the polymer matrix is the key for successful utilization of PLA/silica nanocomposites and to maximize their fit with industrial applications needs.

## 1. Introduction

Owing to its versatility and good performance, polylactic acid (PLA) as a biodegradable polymer is regarded as one of the most favorable polymers to substitute petroleum-based plastics [1,2,3]. The low toughness and poor thermal stability of PLA, however, restrict the usage of PLA in applications, such as food packaging and medical implants [3]. To improve the performance, therefore, several approaches, such as blending with other polymers, mixing with inorganic fillers, and copolymerization, have been suggested by several research groups [4,5,6,7,8,9,10]. In particular, the mixing with inorganic additives, such as TiO_2_, carbon nanotube, ZnO, Al_2_O_3_, MgO, and SiO_2_, was reported to be a useful method to boost the performance of neat PLA [5,7]. 

Silicon dioxide (SiO_2_), commonly referred to as silica, which may exist in the amorphous and crystalline structure, was found to be a useful filler for improving the mechanical performance of polymeric materials [11,12]. The silica, as an additive, is used in coatings, food, and biomedical applications. For example, silica as a bio-safe additive can be incorporated along with silver nanoparticles to improve the antibacterial properties and corrosion properties of biomaterials [13,14,15,16,17,18,19,20,21,22,23]. The amorphous silica nanoparticles extracted from natural sources, such as rice husk and fly ash, can be used as a nucleating agent when added to thermoplastic polymers in very low amounts, owing to their large surface area [24]. The improving dispersibility of silica within the PLA matrix during fabrication of PLA/silica composites would lead to enhancing the mechanical, thermal, and rheological properties of neat PLA [21]. The presence of silanol groups, siloxane bridges, and hydroxyl groups on the silica would facilitate the surface functionalization of silica for biomedical, catalysis, and sensor applications [12]. 

Therefore, by taking into consideration the attractive properties of silica, the fabrication of PLA/silica composites would be a beneficial procedure to enhance the performance of PLA. To the best of our knowledge, this is the first systematic article deliberating the recent progress in PLA/silica. Thus, this work aims to discuss the recent developments in the field of PLA/silica composites in terms of fabrication, properties, and applications. 

## 2. Synthesis of PLA/Silica Composites

The direct melt-mixing, solution mixing, sol–gel process, and in situ polymerization methods are considered as the main methods utilized for the synthesis of PLA/silica composites. The melt-mixing method involves the direct mixing of PLA with silica nanoparticles, while the sol–gel process can be performed either in the existence of PLA or simultaneously during the polymerization of lactic acid monomers [25]. The solution mixing method starts from the dissolution of polymers in a suitable solvent with nanoparticles together with the evaporation of the solvent, or precipitation [25]. As for the in situ polymerization method, the silica nanoparticles should be distributed in the monomers before polymerization [25]. In addition, it is worth mentioning that the freezing-drying process would also be a promising method to fabricate PLA composites. In the freezing-drying process, the colloidal dispersion of the aerogel precursors is frozen, with the liquid component freezing into different morphologies depending on a variety of factors, such as the precursor concentration, type of liquid, temperature of freezing, and freezing container [25]. However, a lack of works was found on the utilization of this method to fabricate PLA/silica composites. 

**Figure 1:**
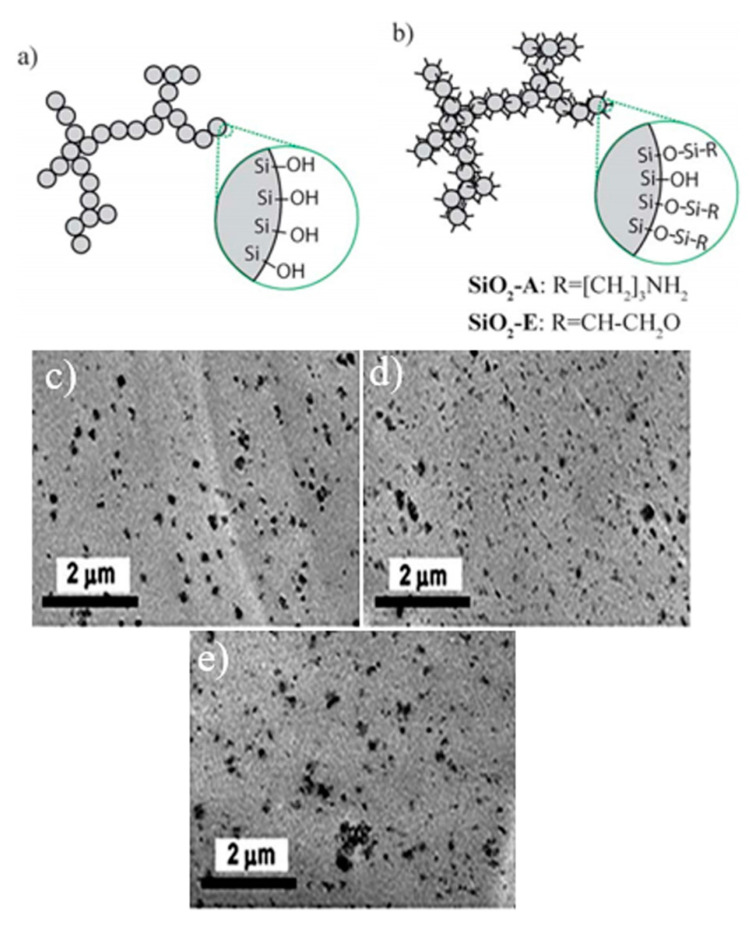
Schematic representation of the (**a**) surface-unmodified silica and (**b**) surface-modified silica aggregate. (**c**–**e**) TEM images of the PLA composites containing (**c**) un-modified SiO_2_, (**d**) SiO_2_-A, and (**e**) SiO_2_-E [26].

The surface modification by physical or chemical methods is a common procedure to increase the compatibility between PLA and silica nanoparticles [26,27,28,29,30]. The functionalization of hydrophilic silica nanoparticles, which is usually conducted on the reactive silanol end-groups, would improve the hydrophobicity of silica nanoparticles, improving their dispersion in the PLA matrix. This process, i.e., the formation of hydrophobic-fumed silica, can be obtained by chemical treatment of hydrophilic silica with silanes or siloxanes. As a result of the improved dispersion of silica particles in the PLA matrix, the rheological properties of the composites are improved. In general, the large surface area and the smooth surface of silica nanoparticles could increase the interactions not only between silica nanoparticles but also between PLA and silica nanoparticles. Thus, good physical interactions between the silica and PLA matrix can be achieved, leading to significant enhancements in composites properties. Several functionalization (coupling) agents, such as tetraethyloxysilane (TEOS) and γ-glycidoxypropyltrimethoxysilanes (GOPTMS) were used to improve the dispersibility of silica nanoparticles in the PLA matrix. In the study of Hakim et al. [26], a reactive extrusion method was utilized for the melt-mixing of PLA with 2.5 wt.% of silica nanoparticles. The schematic illustrations of SiO_2_ before and after surface modification by organic chains are displayed in Figure 1. As shown in Figure 1b, the surface -OH groups of both surface-modified silica types were mostly substituted by functionalized organic chains. Thus, many PLA chains could be grafted on one silica nanoparticle, which would have risen the local shear field applied to the agglomerated nanoparticles during mixing, thereby improving the dispersibility of silica nanoparticles in the polymer matrix. However, transmission electron microscopy (TEM) observations implied that the uniformity and dispersibility of the nanoparticles under the experimental processing conditions were found not to be affected by the enhanced interfacial interaction of both surface-treated silica types (Figure 1c–e). 

The fabrication of PLA/silica composites via the sol–gel method was first reported by Yan et al. [31], who aimed to obtain plasticized composites. The fabrication process of the composites involved an in situ synthesis of silica nanoparticles via condensation reactions of TEOS and GOPTMS in the presence of PLA and polyethylene glycol as a plasticizer in tetrahydrofuran. Hydrochloric acid was utilized as a catalyst during the synthesis method. The infrared results proved the formation of a silica network structure within the PLA matrix, while the results of mechanical tests indicated that the incorporation of 4 wt.% of silica nanoparticles would increase the tensile strength of PLA from 15 to 18 MPa. In another work, Yan et al. [32] polymerized L-lactic acid (PLLA) in the presence of silica without the use of catalysts, but in solution. In this case, the silica surface was not organically treated. The polycondensation was carried out in toluene to remove the water formed by azeotropic dehydration. The result is silica grafted with PLLA oligomers. The authors were able to witness the grafting by infrared spectroscopy characterization (unfortunately, the molar masses are not specified). The grafted silica was then dispersed in PLLA and its good dispersion helped to improve mechanical properties, as compared to the PLLA composites with non-grafted silica.

Wu et al. [32] also polycondensed L-lactic acid in the presence of silica nanoparticles, but this time in bulk. The authors first mixed an aqueous solution of L-Lactic acid (LA) with an acidic silica sol containing silica particles of 12 nm. The mixture was dehydrated under vacuum with sonication treatment to well disperse the particles. After complete drying of the mixture, the polycondensation was performed under vacuum conditions to remove the water formed. The grafting occurred as above, with the polycondensation using the SiOH groups on the surface of the silica nanoparticles. The results showed that the molar mass of the grafted PLLA was about 31,100 g·mol^−1^. Liu et al. [33] fabricated PLA/silica composites through ring-opening polymerization of lactide initiated by modified silica nanoparticles in the presence of stannous octoate as a catalyst. The experimental conditions, such as the weight ratio of silica to the lactide monomer, reaction temperature, and reaction time were optimized to be 1:20, 140 °C, and 72 h, respectively. The morphological observations revealed that silica nanoparticles tended to be distributed uniformly within the PLA matrix, improving compatibility between PLA and silica nanoparticles. Accordingly, the thermal and mechanical properties of the composites were improved significantly in comparison to that of pure PLA. In other works [24,34,35], the melt-mixing method to fabricate PLA/silica composites was carried out at 175 °C. In comparison to neat PLA, the PLA/silica composites containing various contents of silica (from 1 to 10 wt.%) exhibited significant improvements in the thermal stability and the barrier properties against nitrogen and oxygen gases, which were connected with the establishment of the silica network structure as approved by Fourier-transform infrared (FTIR) and rheological results. In the work of Liu and co-workers [36], amino-functionalized nano-SiO_2_ (m@g-SiO_2_) was first prepared through a coupling reaction on the surface of silica nanoparticles before its melt-mixing with PLA. Molecular dynamics simulation was carried out to discover the correlation between PLA and silica nanoparticles before and after the organic functionalization of silica. The crystallization behavior and the mechanical properties of PLA exhibited significant improvements after the melt-mixing with functionalized silica nanoparticles which were attributed to the fact the organic modification of silica nanoparticles would lead to enhancements in the interaction energy and mobility of PLA chains.

To improve the dispersion of silica in the PLA matrix, an in situ melt condensation of l-lactic acid was carried out in the presence of silica nanoparticles [37]. However, when the content of silica exceeded 10 wt.%, some agglomerated nanoparticles appeared in the TEM images of the prepared composites. The agglomeration of silica nanoparticles would be attributed to the strong van der Waals forces, which tended to reduce the physical properties of the obtained composites. Thus, it is believed that the physical mixtures of PLA and organo-modified SiO_2_ resulted in the separation in discrete phases, leading to inferior mechanical properties [38,39]. Zou and coworkers tried to mix silica nanoparticles with a copolymer made of PLA and epoxidized soybean oil [40]. The FTIR and thermogravimetric analysis (TGA) results confirmed the reactions between silica and epoxidized soybean oil, which, in turn, led to improve crystallization behavior and mechanical properties of PLA, while Sepulveda et al. [41] demonstrated that the direct grafting of L-lactic acid oligomer onto the silica surface through its silanol groups was a good strategy to enhance the physical, thermal, and mechanical properties of PLA/silica composites. As such, Zhu et al. [42] found that the surface functionalization of fumed silica nanoparticles oleic acid by oleic acid would help to improve the rheological, thermal, and mechanical properties of PLA/silica composites prepared via the melt-mixing method, which was linked to the good interfacial adhesion in the composites containing functionalized-silica nanoparticles.

## 3. Rheological Properties

**Figure 2:**
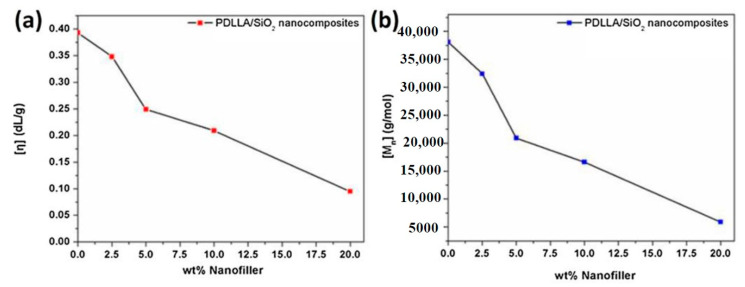
Effect of silica content on the (**a**) intrinsic viscosity and (**b**) average molecular weight of PDLLA [52].

Understanding the rheological properties is very important due to their considerable effects on molecular weight, morphology, chain structure, and chain motions [43,44,45,46,47,48,49]. Basilissi et al. [50] demonstrated that the melt viscosity of PLA/silica composites fabricated by bulk ring-opening polymerization can be improved by the silane-based modification of silica nanoparticles. Li et al. [51] reported that both the storage modulus and biodegradation rate of PLA tended to be improved by the addition of silica nanoparticles into the PLA matrix. The formation of a silica network structure was responsible for storage modulus improvements, while the enhancement in the biodegradation rate was ascribed to the easy release of silica aggregates from the PLA matrix. Nerantzaki et al. [52] prepared a series of poly(DL-lactide) (PDLLA)/SiO_2_ composites by a novel two-step technique (ring-opening polymerization (ROP)—polycondensation). The concentration of SiO_2_ was varied from 2.5 to 5, 10, and 20 wt.%. The results of this work are presented in Figure 2. Based on the intrinsic viscosity results, it was demonstrated that the average molecular weight of PDLLA (Mn ≈ 38,097 g mol^−1^) tended to reduce with an increase in the silica content. Moreover, the average Mn values of PLA were found to decrease when the content of silica increased from 2.5 to 20 wt.%. This finding was elucidated based on the fact that the addition of silica to the PDLLA matrix would hinder the increment in the molecular weight of the polymer. Besides, silica nanoparticles can interact with DL-lactide through their silanol groups. In another study [53], the silica was functionalized with TEOS and GOPTMS through graft-condensation reaction. Afterward, the functionalized silica was melt-mixed with PLA by reactive extrusion technique. In comparison to neat PLA, the addition of functionalized silica to the PLA matrix caused considerable improvements in values of the complex viscosity and the storage modulus of the PLA/silica composites. The inclusion of silica was expected to enhance the hydrolysis resistance by the formation of a stable silica network. Although the molecular weight of PLA was reduced by 12 wt.% during the processing, a slighter reduction of less than 10% was reported upon the addition of TEOS, which also resulted in increasing the value of zero-shear rate viscosity (η_0_) obtained via the Cross model. 

**Figure 3:**
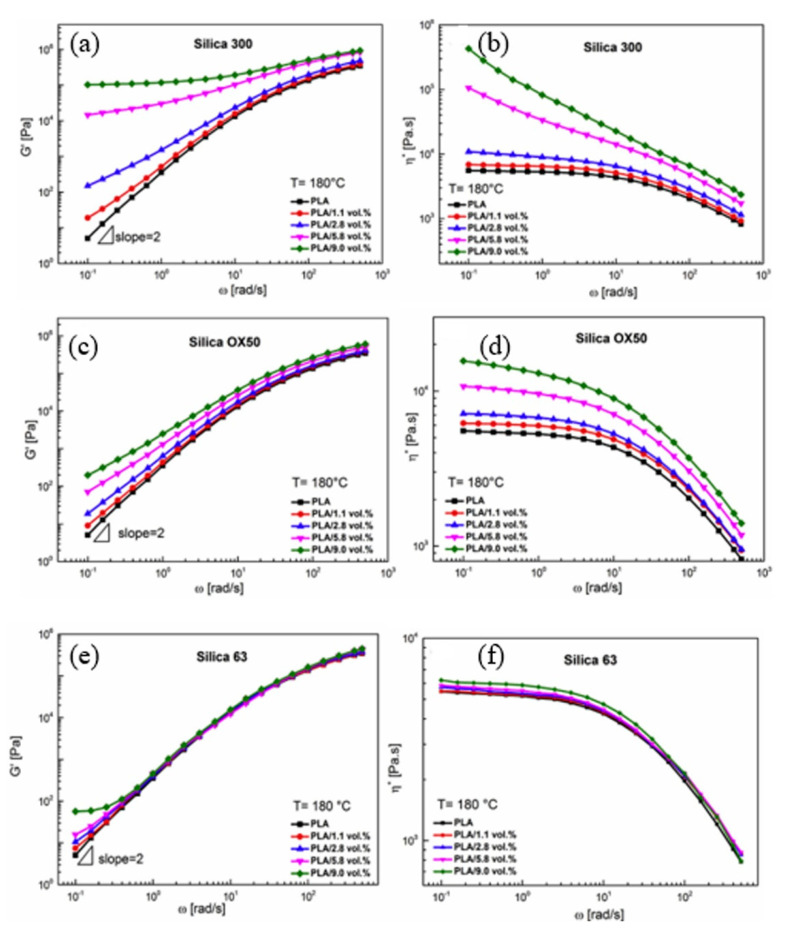
The influence of silica type on the rheological properties of PLA/silica composites: (**a**,**b**) PLA/silica 300; (**c**,**d**) PLA/silica OX50; (**e**,**f**) PLA/silica 63 [54].

As reported by Hao’s group [54], the inclusion of 1.1, 2.8, 5.8, and 9.0 vol.% of silica particles into the PLA matrix led to the improvement in the viscoelastic properties of PLA. Here, silica with different sizes (same density), namely, silica 300 (7 nm), silica OX50 (40 nm), and silica 63 (9000 nm), were used. As shown in Figure 3, the low-frequency G’ increased with the addition of silica and reached an approximately frequency-independent plateau at the high content (above 2.8 vol.%). Moreover, it was observed that the effect induced by silica 63 on the storage modulus (G0) and complex viscosity (η*) of PLA/silica 63 composites was extremely weak. With the increased silica loading, G0 varied negligibly in the high-frequency region and just a slight increase was found at the low-frequency range. Moreover, the times obtained from the plots of storage modulus (G′(t)/G′ onset versus time) at 180 °C in the case of neat PLA were found to be 5000 s, while longer times of 8000 s were reported for PLA/silica composites. The results obtained in this work clearly show that the rheological properties of PLA/silica composites are strongly affected not only by the particle size but also by the particle content. 

## 4. Thermal Properties

**Figure 4:**
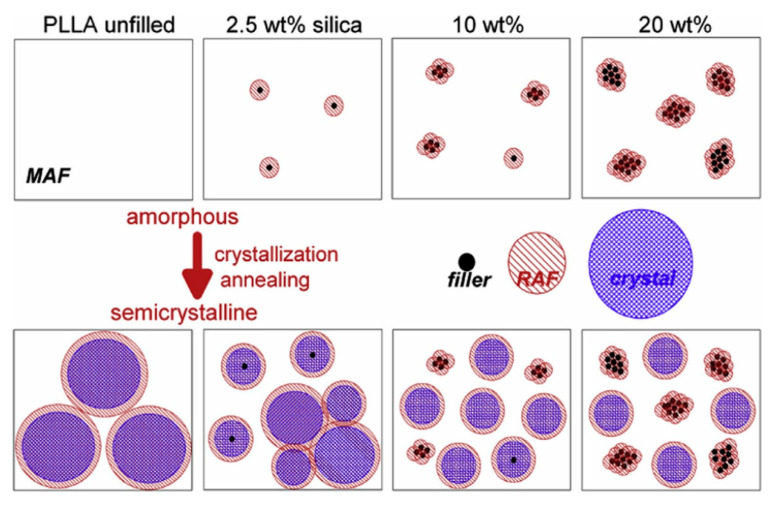
The proposed distribution of the silica in the PLA matrix before and after annealing of crystallization [57].

The thermal stability of PLA can be improved by the incorporation of silica nanoparticles. In the works of Wen et al. [24,55], PLLA/silica composites were fabricated via a melt-mixing method. The thermal stability of PLA showed significant improvement with the addition of silica which was linked to the barrier effect of the silica network structure, which was also responsible for the improvements in the rheological properties of PLA discussed in the previous chapter. Zhang et al. [56] reported experimentally and theoretically, using a molecular dynamics simulation, that the thermal properties of PLA tended to be enhanced with the addition of silica into the polymer matrix during the processing in a twin-screw extruder. The results indicate that the glass transition temperature (T_g_) of PLA was increased by 1.34 °C, while the thermal stability was increased by 12 °C when 2 wt.% of fumed silica was added into the PLA matrix. Klonos and Pissis [57] examined the thermal behavior of PLA/composites by taking the role of H-bonds, formed due to the reaction between the carbonyl groups in PLLA with hydroxyl groups on the silica surface, into account. Examinations of findings involved a combination of assessments on initially amorphous and on semicrystalline (annealed) samples. No change in the T_g_ by the silica was noted by differential scanning calorimetry (DSC), whereas the heat capacity step decreased in the PNCs. The segmental relaxation in the broadband dielectric relaxation spectroscopy (DRS) became, however, quicker and weaker in the PLA/silica composites. They reported that T_g_ and the temperature difference between onset (T_onset_) and end (T_end_) of the event (DΤ_GT_ = T_end_ + T_onset_) tended to increase after annealing of crystallization, by 6–8 K and by a factor of ~2, respectively, as compared to the amorphous samples, due to constraints imposed by crystallites and heterogeneities. As for semicrystalline composites, T_g_ was increased by ~2 K, as compared to neat PLA. Simultaneously, DΤ_GT_ decreased slightly with increasing silica content. The authors attributed these results to the changes in semicrystalline morphology (Figure 4), polymer diffusion, and porosity/dispersion of silica particles.

In the work of Bouamer et al. [58], a casting method was used to fabricate hybrid PLA composites with silica and AlO particles. In this regard, 10 wt.% of either silica or AlO particles was incorporated into the PLA matrix to fabricate PLA/silica and PLA/AlO composites, respectively, while 5 wt.% of SiO_2_ and 5 wt.% of AlO particles were added to the PLA matrix to formulate PLA/silica/AlO composites. Based on the XRD patterns, it was reported that the crystallinity of the PLA acid film could be increased with the addition of silica/AlO particles into the PLA matrix. This result was attributed to the nucleating role of silica and AlO particles, which would facilitate the crystallization of PLA. However, Santos et al. [59] reported that the crystallinity of PLA was not affected by the inclusion of silica into the PLA matrix, while the dual addition of silica and cellulose into the PLA matrix resulted in significant improvements in the crystallization behavior of PLA. This finding was ascribed to the synergism between the two types of nanoparticles in which the agglomeration of cellulose nanoparticles could be prevented in the presence of silica. Thus, the nucleation was related not only to the chemical nature of the particles but also to the increased contact surface between the silica and cellulose nanoparticles. Zou et al. [60] demonstrated that the inclusion of a proper amount of silica nanoparticles (nucleating agent) would cause a reduction in the nucleation barrier shortening the nucleation period of PLA. The higher crystallinity was obtained in the composites containing 1.5 wt.% of silica nanoparticles. Such an amount of silica was expected to increase the interfacial compatibility and crystallinity of the composites thus enhancing the thermal stability. According to Prapruddivongs et al. [61], the crystallinity of the PLA film would have been affected by the type of silica added to the PLA matrix. Here, two types of silica, such as commercial silica (CSiO_2_) and silica extracted from rice husks (RSiO_2_), were added. The DSC thermograms of PLA composites (containing Triallyl isocyanurate and dicumyl peroxide) indicated that the silica nanoparticles affected the crystallinity and the melting behavior of PLA by impeding the chemical crosslinking reactions which were reflected by the change in the FTIR functional band of silica/CPLA composites at 1685 cm^−1^.

Prapruddivongs and coworkers [62] studied the properties of PLA/silica and chemically crosslinked PLA (CrPLA)/silica composites prepared via the melt-mixing method in the presence of triallyl isocyanate and dicumyl peroxide as crosslinking agents. Here, two types of silica, such as CSiO_2_ and RSiO_2_, were used. Irrespective of the silica type, the thermal properties of the PLA/silica CrPLA/silica composites were improved. The addition of 1 wt.% of CSiO_2_ and RSiO_2_ led to an increase in the T50 value (the temperature associated with loss of 50 wt.%) of PLA from 321 °C to 339 °C and 342 °C, respectively. It was found also that the degradation temperature of PLA composites tended to increase in the presence of triallyl isocyanate and dicumyl peroxide. Moreover, the thermal stability of CrPLA/RSiO_2_ composites was better than the thermal stability of CrPLA/CSiO_2_, implying that RSiO_2_ was an efficient additive for enhancing the thermal stability of PLA and CrPLA. Vidakis and coworkers [63] prepared PLA/silica composites via the melt-mixing of PLA with different contents of silica, such as 0.5, 1, 2, and 4 wt.%. Although the mass loss for the PLA/4 wt.% silica composite was lower than for PLA/1 wt.% silica, the overall thermal stability of the PLA seemed not to be influenced by the existence of the silica filler, which would be related to the strong H-bond interactions between Si–OH in silica nanoparticles with PLA chains. This is, in turn, restricted their release into the environment. 

**Figure 5:**
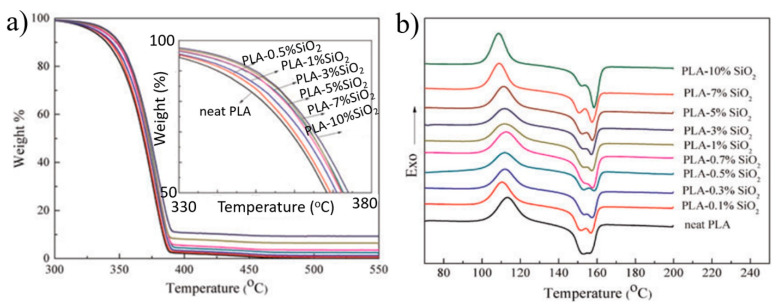
The effects of SiO_2_ content on the thermal behavior of neat PLA and PLA/silica composites: (**a**) thermogravimetric analysis; (**b**) crystallization and melting behavior [64].

Lv et al. [64] examined the thermal properties of the PLA/silica composites prepared via the melt-mixing method. As displayed in Figure 5a, the increase in the silica content increased the degradation temperature of PLA/silica composites. For example, the inclusion of 10 wt.% of silica led to increasing the degradation temperature by 15 °C, indicating a significant increase in the thermal stability of PLA was achieved. From Figure 5b, it was found that the cold crystallization temperature (T_cc_) of neat PLA acid tended to be shifted to lower temperatures upon the inclusion of silica nanoparticles, suggesting that plying the silica nanoparticle worked as nucleating agents or it could hinder crystallization from the melt. While the low-temperature melting peaks became weak, the high-temperature melting peaks showed a gradual increase with the increase in the silica content in the composites. This result would suggest that the inclusion of silica causes a reduction in the defective crystals, increasing the perfect crystals for the PLA phase. According to Ge et al. [65], the dispersibility of silica nanoparticles in the PLA matrix could be improved when the content of silica was less than 3 wt.%. Accordingly, the crystallinity of PLA was improved. The best crystallization behavior was obtained when 1 wt.% of silica was added to the PLA matrix. However, although some agglomerated nanoparticles were observed, the thermal stability of PLA was enhanced by the addition of silica nanoparticles.

The effect of silica content on the T_g_ of PLA/silica composites was explored by Pilić and coworkers [66]. It was reported that the addition of low amounts of silica, such as 0.2 and 0.5 wt.%, into the PLA matrix led to an increase in the T_g_ of neat PLA (47.6 °C) to 48.9 and 50.6 °C, respectively. This behavior was ascribed to the fact that the chain mobility throughout the PLA matrix volume tended to be decreased in the presence of silica nanoparticles, while the high loadings of silica, such as 1, 2, 3, and 5 wt.%, resulted in a decrease in the values of T_g_ in comparison to that in neat PLA, which was assigned to the agglomeration of silica nanoparticles within the PLA matrix which affected the chain mobility of the polymer. However, Wen et al. [56] reported that the addition of various loadings of silica, such as 1, 3, 5, 7, and 10 w.%, did not affect the value of T_g_ of PLA, suggesting that the impacts of silica on the chain mobility of PLA were insignificant. 

Techawinyutham et al. [67] examined the thermal stability of PLA and porous silica-containing capsicum oleoresin (SiCO)-modified PLA composites before and after the accelerating weathering test. The results imply that the accelerated weathering test induced the photolysis and partial hydrolysis, which resulted in improvements in the crystallization behavior of PLA. In addition, the accelerated weathering reduced the storage and loss moduli due to the increased chain mobility caused by the chain scission of the polymer chains. 

Wu et al. [68] first grafted PLA on the surface of silica nanoparticles, then examined the thermal properties of PLA/PLA-grafted silica composites. Based on the DSC results, it was found that the addition of PLA-grafted silica could accelerate the crystallization rate of PLA. Besides, PLA/PLA-grafted silica composites exhibited typical homopolymer-like behavior in the final structure, regardless of the PLA-grafted silica content. The PLLA/silica composites containing 2.5 wt.% of silica exhibited a shielding effect to the evolution of gases that were released during the decomposition, improving mostly the initial stages of thermal degradation [69]. The thermal stability of PLA could be increased by 20 °C when low amounts of stearic acid-modified silica nanoparticles (0.1–1.5 wt.%) were added to the PLA matrix [40]. Similarly, Khankrua et al. [70] demonstrated that the presence of 5 wt.% of silica nanoparticles in the polymer matrix could cause significant improvements in the thermal stability of PLA. The effect of untreated silica nanoparticles on the thermal stability of PLA was examined by Basilissi et al. [51]. They found that the thermal stability of the composites tended to improve with the increase in the silica content. For instance, the temperature corresponding to 5 wt.% weight loss in the case of PLA composites containing 2 wt.% silica was higher by 70 °C than the counterpart corresponded to neat PLA, which was connected to the formation of silica network structure in the composites. The formation of a silica network structure hindered the diffusion of volatile decomposition products out of PLA and the diffusion of oxygen into the matrix. Similar results were reported by Wen et al. [24], who fabricated PLA/silica composites via the melt-mixing method and found that the thermal stability of PLA could be increased by the addition of ≤5 wt.% silica. However, the higher loading of silica (≥5 wt.%) would cause an agglomeration of the nanoparticles in the polymer matrix due to the weak dispersion, which led to a reduction in thermal stability of PLA. In contrast, Lv et al. [64] found that the thermal stability of PLA/silica composites made by melt-mixing method tended to increase with the increase in the silica content—even when high loadings of silica, such as 7 and 10 wt.%, were added to the PLA matrix. Similarly, Mustapa et al. [71] postulated that the thermal stability of PLA could be increased with the addition of 2.5 and 7.5 wt.% of silica nanoparticles. In another works by Mustapa and coworkers [72,73,74], it was reported that the melting temperature and crystallization behavior were affected by the addition of silica nanoparticles, which acted as nucleation sites that induced the crystallization phenomenon of PLA. Lai and Li [75] functionalized the silica by the melt-mixing with polyurethane and found that the functionalized silica could greatly trigger the nucleation and crystallization behavior of PLA, when compared to the counterpart composites with non-functionalized silica. The influence of silica particles on the thermal properties of PLA composites is summarized in Table 1. 

## 5. Mechanical Properties

Several research teams have aimed to improve the mechanical properties of PLA with the addition of silica to the polymer matrix. For example, He et al. [76] reported that the inclusion of silica nanoparticles is a good strategy to improve the mechanical properties of PLA. Indeed, the tensile strength (TS), Young’s modulus (YM), and impact strength of the PLA/silica composites made by the melt-mixing method were greatly increased with the addition of silica nanoparticles, which was linked to the good dispersibility of the particles in the polymer matrix, as well as PLA-silica interactions [77]. The low loading of silica (below 5 wt.%) was found to be distributed homogenously within the PLA matrix, while some agglomerates were observed at the high loading (higher than 5 wt.%) [36]. Ahmed et al. [78] studied the impact of silica additive into PLA matrix-3D printing. When the silica addition amount was 10 wt.%, its TS, flexibility, YM, and other properties were greatly enhanced. The application of this kind of composite material is feasible, but once the silica content exceeds 15%, the performance is reduced. The improved mechanical properties promote the recycling of PLA while retaining its biodegradability. As such, YM and ultimate tensile stress (UTS) of PDLLA were increased by 106% and 63.7%, respectively, upon the addition of 3 wt.% silica into the polymer matrix [79].

Yan et al. [32] prepared PLA/silica composites via two steps: grafting of L-lactic acid oligomer onto the silica surface, followed by melt-mixing with PLA. This procedure led to fabricating composites with improved mechanical properties. In another work by Yan and co-workers [31], the TS of the plasticized PLA/silica composite could be increased to large values even in the presence of low loadings of silica. Although the addition of the silica nanoparticles accelerated the biodegradation rate of PLA, the resultant composites had improved mechanical properties, as reported by Georgiopoulos et al. [80].

**Figure 6:**
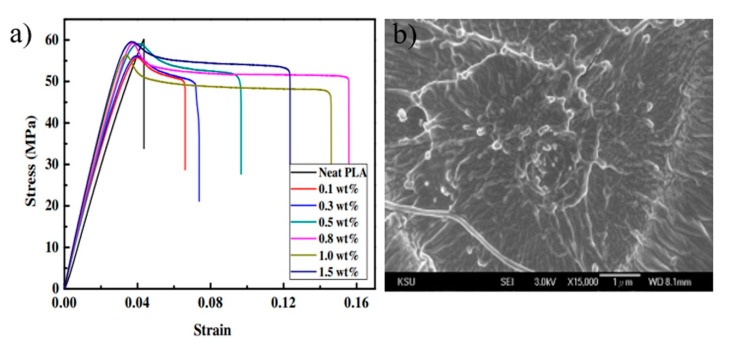
(**a**) The stress–strain curve of neat PLA and PLA composites. (**b**) The fracture morphology after the tensile test of PLA composite [38].

To obtain better dispersion in the PLA matrix, silica nanoparticles with 24 nm in average diameter were functionalized via stearic acid [38]. Different contents of silica, such as 0, 0.1, 0.3, 0.5, 0.8, 1, and 1.5 wt.%, were mixed with PLA via an extrusion technique. The mechanical tests revealed that the addition of 0.8 wt.% of silica nanoparticles into the PLA matrix led to increasing the Izod impact strength from 1.57 to 5.13 kJ/m^2^. The impact strength values of the PLA/silica composites increased with increasing silica content, then decreased when the silica content exceeded 0.8 wt.%. Moreover, the presence of nano-silica in the PLA matrix caused improvements in the EB and TS of PLA (Figure 6a). As shown in Figure 6b, dimple morphologies were observed on the fractural surfaces of PLA/silica composites, suggesting that neat PLA was less ductile than PLA/silica composites. According to the lubrication sliding and the ductile behavior, it was confirmed the toughness of PLA could be largely improved by the incorporation of silica nanoparticles. The effects of silica content (1, 3, and 5 wt.%) on the mechanical performance of PLA film were studied Zirak and Tabari [81]. It was found that the values of the TS and YM of PLA tended to be increased upon the incorporation of silica nanoparticles into the PLA matrix, regardless of the silica content. For example, the addition of 5 wt.% of silica into PLA led to an increase in the values of TS and YM for PLA from 29 MPa and 2.3 GPa to 43 MPa and 3.1 GPa, respectively, which was attributed to the reinforcing effect of SiO_2_ nanoparticles. In contrast, the value of EB of PLA (17.5%) was reduced with the inclusion of silica nanoparticles, where a value of 10.5% was found in the composite containing 5 wt.% SiO_2_. 

In Yan’s research [32], it was approved that the grafting of L-lactic acid oligomer on the silica nanoparticles by condensation reaction without catalyst would result in the formation of a good filler, helping to increase the toughness of PLA. Chrissafis et al. [82] reported that the inclusion of 2.5 wt.% of fumed silica and montmorillonite into the PLA matrix would result in the formation of composites having higher values of TS and YM than neat PLA. However, the addition of fumed silica and montmorillonite to PLA led to a reduction in the value of EB, implying that those two additives acted as reinforcing agents. Lai and Hsieh [83] examined the influence of the surface functionalization of silica on the mechanical properties of PLA. To form the modified silica, polyethylene glycol methyl ether was grafted on the silica in the presence of aminosilane. The interfacial interaction between silica and PLA was improved in the presence of modified silica; thereby, the value of TS of the composites with modified silica was higher than the counterpart composites containing unmodified silica. 

Battegazzore et al. [84] used different contents, such as 5, 10, 20, and 30 wt.%, of silica powder, obtained by the conventional extraction method from rice husk, to fabricate PLA/silica nanocomposites via the melt-mixing method. Based on Archimedes law, the silica density was calculated to be 1.82 and 1.88 g·cm^−3^ for the extracted silica and commercial silica, respectively, while the density of PLA was 1.25 g·cm^−3^. The density values were used to calculate the volumetric fractions. When the extracted silica nanoparticles were melted with PLA, the YM of neat PLA was increased, while the oxygen permeability of PLA was slightly reduced. As compared to the composites including 10 and 30 wt.% of commercial silica, the counterpart composites with extracted silica exhibited better mechanical properties. Considering the economic analysis of the whole process and by reusing the energy recovered from burning rice husk, the authors considered the composites containing 20 wt.% of extracted silica as economically sustainable materials. 

It is worth mentioning that silica can improve the mechanical properties of other PLA composites. For example, the addition of silica can improve the jute/PLA interfacial adhesion, which results in the enhancement of the mechanical performances of the resultant composites [85]. 

On the other hand, the influence of other silica-based materials on the properties of PLA was explored by many research groups. For instance, the addition of glass fibers to the PLA matrix improved the mass flow rate and flexural modulus, while their effects on the impact and flexural strength, as well as thermal stability of PLA, were insignificant [86]. Besides, the TS and EB were reduced with the addition of glass fibers.

## 6. Biodegradbility and Other Properties

**Figure 7:**
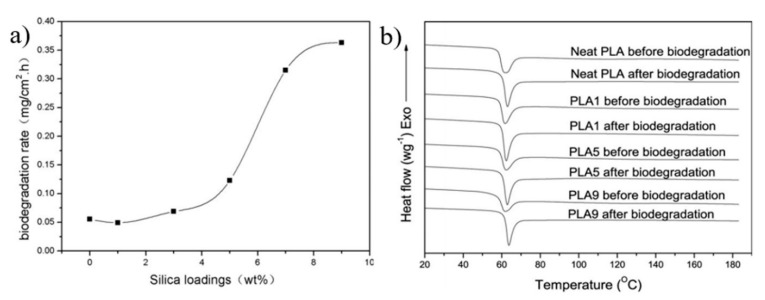
(**a**) The effect of silica content on the biodegradation rate of PLA/silica composites. (**b**) The DSC curves of the PLA/silica composites before and after degradation of 24 h [50].

In general, significant research still needs to be performed to achieve the final target of ideal biodegradable PLA/silica composites that exhibit high performance and easy biodegradability when their roles are completed. The hydrophilic nature of silica nanoparticles is expected to affect the degradation of PLA. The hydroxyl groups in silica are bound together by hydrogen bonds and can assist the hydrogen bonding interaction with the functional groups in PLA or a covalent bonding with a macromolecular chain [87]. Thus, silica nanoparticles are expected to facilitate the hydrolysis or enzymatic attacks of ester groups of PLA, leading to a fast biodegradation rate. For instance, Li et al. [50] reported that the weight loss during the biodegradation process of PLA/silica composites, fabricated by melt-mixing method, was larger than that in neat PLA. Indeed, the incorporation of 9 wt.% of silica nanoparticles into the PLA matrix led to a biodegradation rate of 0.36 mg cm^−2^ h^−1^, which was 6.5 times higher than that of neat PLA. Figure 7a shows the fast biodegradation of the PLA composites compared to neat PLA. This result was confirmed by the DSC curves shown in Figure 7b. As shown in Figure 7b, all samples were amorphous at a T_g_ of 60 °C. The improvements in the degradation rate were attributed to the easy release of silica particles from the PLA matrix. The hydrophilic silica facilitated the hydrolysis and enzymatic attack of ester groups of PLA chains. The biodegradation was more pronounced in the composite membrane after 2 months of in vitro tests [88]. On the other hand, it was reported that the flame retardant properties of PLA could be improved greatly by adding treated silica nanoparticles into the PLA matrix [64]. Indeed, the weight loss ratios of PLA/silica composites at different burning time intervals tended to decrease with the increase in the silica content, which was linked to the excellent dispersion of particles in the PLA matrix. So, the larger specific surface area in the composites provided a better effect of thermal insulation [64]. SiO_2_-fluorinated PLA composites can be used as reversible and highly hydrophobic coatings to protect the exterior of buildings [89]. Similarly, it was reported that the flame retardancy of PLA could be improved by the addition of fumed silica and Ni_2_O_3_ into the polymer matrix [90].

**Figure 8:**
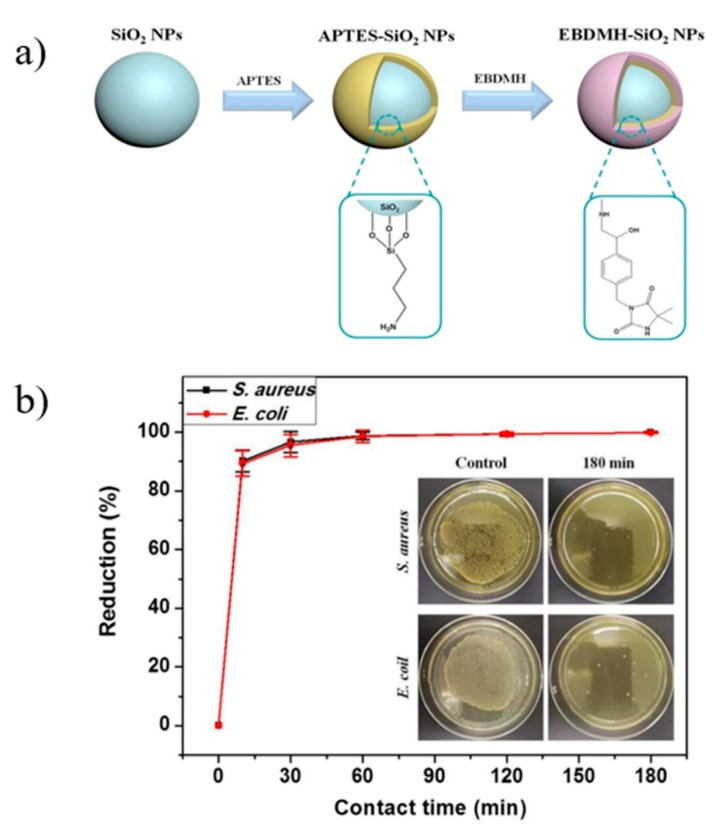
(**a**) Synthesis of *N*-halamine precursor-modified silica nanoparticles (EBDMH–SiO_2_ NPs). (**b**) Antibacterial tests of PLA/EBDMH–SiO_2_-9 plastic sheets after chlorination against *S. aureus* and *E. coli*. In the inset are photographs showing the bacterial culture plates of *S. aureus* and *E. coli* upon 180 min contact with the control and PLA/EBDMH–SiO_2_-9 plastic sheets [91].

A study has been reported by Zhao et al. [91], where *N*-halamine precursor with epoxy and hydantoin structures, 3-(4′-epoxyethyl-benzyl)-5, 5-dimethylhydantoin (EBDMH) was utilized for the *N*-halamine-modified silica nanoparticles. EBDMH was fabricated and immobilized onto aminofunctionalized silica nanoparticles to form EBDMH–SiO_2_ nanoparticles (Figure 8a). PLA was mixed with EBDMH–SiO_2_ nanoparticles via the melt-mixing method. The efficiency of the PLA/EBDMH–SiO_2_ composite as antimicrobial material was evaluated against *S. aureus* and *E. coli*, respectively, and the obtained results are shown in Figure 8b. The PLA/EBDMH–SiO_2_ composite exhibited excellent bactericidal efficiency. Indeed, the PLA/EBDMH–SiO_2_ composite with a contact time of 10 min neutralized about 90.2% of *S. aureus* and 89.4% of *E. coli*. When the contact time was increased to 180 min, a kill efficiency of 99.97% and 99.91% against *S. aureus* and *E. coli*, respectively, was obtained. Due to improved biocompatibility between PLA and silica, as well as the excellent antibacterial efficiency, the PLA/EBDMH–SiO_2_ composite could be utilized for hygienic product packaging and filters, as well as medical textiles.

The impact of silica nanoparticles on the interfacial tension between PLA and supercritical CO_2_ at high temperature and high pressures was examined by Sarikhani and coworkers [92]. The addition of a low loading of silica (less than 2 wt.%) led to reducing the interfacial tension, while the interfacial tension tended to increase when the silica content was larger than 2 wt.%, which was linked to the fact that higher levels of silica originated attractive lateral capillary forces due to the perturbation of the PLA–CO_2_ interface by particles. The interfacial interactions between PLA and silica nanoparticles were found to be decreased with an increase in the CO_2_ content, which facilitated the adsorption behavior at higher pressures. Based on the experiments carried out by Seng and co-workers [93], it was reported that the addition of 1 wt.% of silanol treated-silica into the PLA matrix led to a decrease of 40% in the hygroscopicity of PLA, while the other loadings of silica caused improvements between 3 and 19% in the reduction in hygroscopicity. Chen et al. [94] reported that the hydrophilicity of PLA/silica composites tended to be improved with increasing the silica content, where the inclusion of 5 wt.% of silica into the PLA matrix led to a decrease in the water contact angle of PLA from 82° to 68°. In other words, the less silica in the composite, the larger the contact angle is, thereby, the higher the hydrophobicity of the composite surface is. Thus, the hydrolytic degradation ability of PLLA/silica composites was accelerated in the presence of higher loadings of silica in the polymer matrix.

Kosowska and Szatkowski [95] examined the impacts of silica addition on the ultraviolet aging of PLA nonwovens obtained by the electrospinning technique. The inclusion of silica nanoparticles greatly increased the percentages of crystalline and amorphous phases in the fabricated films and altered the photodegradation mechanism. In the work of Jin-Bo [96], a novel porous membrane composed of PLA with the addition of SiO_2_–CaO by the sol–gel method was designed. The surface potentials of the composite membranes became more negative with higher SiO_2_–CaO contents. Apatite with an orderly ring structure nucleated and grew on the surface of the composite membranes after immersion in SBF for 7 days, implying that the incorporation of SiO_2_–CaO significantly improves the bioactivity of PLA. Based on the dielectric spectroscopy study [97], it was reported that the lifetime and thermal stability of the electric state in PLA can be increased with the addition of SiO_2_, where the optimal concentration of silica for negative corona electrets was found to be 4 wt.%. 

## 7. Potential Applications of PLA/Silica Composites

Owing to its high resistance to heat and good thermal stability, silica has received considerable attention in 3D-printing applications. The enhanced melt viscosity of PLA/silica composites could facilitate the molding and processing of these materials which could lead to a variety of applications [54]. Since the usage of PLA used in the bone tissue applications would not be able to withstand the high-load resistance associated with such applications, the incorporation of silica would make PLA a suitable candidate in these applications, as the flexural modulus and the tensile strength of PLA tended to be improved significantly with the incorporation of a silica filler. For this reason, it was reported that the biorenewable PLA/SiO_2_ composites can be used as promising materials in the bone repair processes in animal models [98]. As indicated by cell-culture measurements conducted by Abe et al. [99], PLA/silica composite could exhibit excellent cytocompatibility. Interestingly, the ROS-responsive LDLR peptides-conjugated PLA-coated mesoporous silica nanoparticles were reported to have an important opportunity for oxidative stress therapy in the central nervous system [100]. Moreover, the good thermal stability and mechanical properties of PLA/silica composites would make these materials suitable for structural applications where high thermal stability and high mechanical strength are needed [71]. Thanks to their physical, thermal, rheological, and mechanical properties, the PLA/silica composites can be used in 3D-printing applications, as indicated by Thongsang and co-workers [101]. Moreover, the PLA/silica composites have a great opportunity to be used as smart and active packaging. According to Jaikaew [102], the CO_2_/O_2_ permeability ratio of PLA/silica composite films can be tuned by varying the types of silica particles and their compositions. Light transmission reduction in both the UV and visible regions is achieved in PLA/modified-silica bio-composite films. As approved by Opaprakasit et al. [103], the films obtained from PLA/modified silica composites have strong potential to be used as biodegradable packaging materials with tunable gas permeability.

## 8. Future Outlooks of PLA/Silica Composites 

The state of knowledge discussed above clearly shows that the fabrication, properties, and applications of PLA/silica composites will keep growing in the future. We thought that future trends of the research on PLA/silica composites should be focused on several relatively new directions, as follows:

- Although PLA/silica composites have been utilized in 3D-printing applications, more research would be needed in this area, as the main challenge in 3D-printing applications is enhancing the flexibility, as well as controlling the viscosity, of PLA.
- The dispersion of silica nanoparticles in the PLA matrix should be improved, as various undiscovered applications in various fields, such as the aerospace, energy, and chemical industries, are expected to be available shortly.
- The utilization of PLA/silica composites to produce hierarchically porous PLA materials for biomedical applications, such as tissue scaffolding, would be a promising research area shortly. The processing conditions, however, should be selected carefully to successfully fabricate such materials.
- Even PLA/silica composites exhibited some levels of biodegradability; they are still far from being considered a solution for plastic waste accumulation.

## 9. Conclusions 

Based on the brief discussion reported in this work, the inclusion of silica nanoparticles into the PLA matrix is one of the promising methods to enhance the performance of PLA while retaining its biodegradability. The fabrication methods, namely, melt blending and in situ polymerization, as well as the organo-modification of silica nanoparticles to increase their affinity to hydrophobic PLA, would play a pivotal role in the final performance of PLA/silica composites. Indeed, the surface functionalization of silica nanoparticles had critical impacts on the rheological performance of the silica within the polymer matrix. The thermal stability, biodegradability, YM, and TS of PLA were greatly improved by the inclusion of silica nanoparticles into the polymer matrix. Such improved properties qualified the PLA/silica composites to be suitable candidates in food packaging, 3D printing, and biomedical applications. 

## Figures and Tables

**Table 1:** The influence of silica incorporation on the thermal properties of PLA composites.

Silica (wt.%)	T_g_ (°C)	T_cc_ (°C)	T_onset_ (°C)	T_max_ (°C)	∆H_m_ (J/g)	Ref.
10	-	-	342.3	370.9	-	[24]
5.0	59	135	-	-	8	[35]
2.0	64.5	0	-	-	31.32	[36]
0.5	52.3	102.8	113.5	-	54.3	[49]
3.0	61.22	107.87	162.60	168.70	34.57	[55]
2.0	52.23	-	-	-	-	[56]
2.5	60	-	-	-	37	[57]
1.5	-	-	-	300	-	[60]
4.0	60.8	108.3	146.6	153.6	24.9	[61]
4.0	-	-	273	374	-	[63]
10	-	-	-	370	-	[64]
5.0	-	120	-	-	70	[65]
0.5	50.6	-	-	-	-	[66]
3.08	57.66	112.33	147.5	153.41	22.73	[67]
0.5	61.02	124.26	-	-	-	[68]
0.2	-	-	345.54	362.56	-	[70]
7.5	59.0	-	355.5	412.3	-	[71]
7.5	59.0	124.94	-	-	6.22	[72]
7.5	60.0	96.3	355.5	412.3	9.9	[73]
7.5	57.4	117	-	-	-	[74]

## Data Availability

The data presented in this study are available on request from the corresponding author.
